# GATA-4-expressing mouse bone marrow mesenchymal stem cells improve cardiac function after myocardial infarction via secreted exosomes

**DOI:** 10.1038/s41598-018-27435-9

**Published:** 2018-06-13

**Authors:** Ji-Gang He, Hong-Rong Li, Jin-Xiu Han, Bei-Bei Li, Dan Yan, Hong-Yuan Li, Ping Wang, Ying Luo

**Affiliations:** 1 Department of Cardiac and Vascular Surgery, First People’s Hospital of Yunnan Province, No. 157 Jinbi Road, Kunming, Yunnan Province 650032 China; 2 Kunming University of Science and Technology, NO.68, Wenchang Road, 121 Street, Kunming, Yunnan Province 650032 China

**Keywords:** Physiology, Cardiovascular biology

## Abstract

This study aimed to investigate whether exosomes secreted by mouse GATA-4-expressing bone marrow mesenchymal stem cells (BMSCs) could induce BMSC differentiation into myocyte precursors, decrease cardiomyocyte apoptosis, and improve cardiac function following myocardial infarction (MI). BMSCs were transduced with a lentivirus carrying a doxycycline (DOX)-inducible *GATA-4* or control lentivirus, and secreted exosomes from these BMSCs were collected and co-cultured with BMSCs or cardiomyocytes under hypoxic and serum free conditions. Furthermore, exosomes were injected into mice 48 h after MI. Cardiac function was evaluated by echocardiography at 48, 72, and 96 h after exosome treatment. Quantitative PCR showed that co-culture of BMSCs with GATA-4-BMSC exosomes increased cardiomyocyte-related marker expression. Co-culture of GATA-4-BMSC exosomes with cardiomyocytes in anoxic conditions decreased apoptosis as detected by flow cytometry. Injection of GATA-4-BMSC exosomes in mice 48 h after MI increased cardiac function over the next 96 h; increased cardiac blood vessel density and number of c-kit-positive cells and decreased apoptotic cardiomyocyte cells were also observed. Differential expression of candidate differentiation- and apoptosis-related miRNAs and proteins that may mediate these effects was also identified. Exosomes isolated from GATA-4-expressing BMSCs induce differentiation of BMSCs into cardiomyocyte-like cells, decrease anoxia-induced cardiomyocyte apoptosis, and improve myocardial function after infarction.

## Introduction

Myocardial infarction (MI) is a major cause of death and disability. In recent years, bone marrow mesenchymal stem cell (BMSC) transplantation has been used as a reparative therapy after MI. BMSCs can differentiate into cardiomyocytes or endothelial cells^1^ and also have anti-inflammatory effects^2^, which are potentially useful for regeneration of cardiac tissue, angiogenesis, and prevention of ischemia/reperfusion damage. However, both cost and transplant rejection for BMSCs represent potential hurdles to the widespread adoption of this therapeutic strategy.

Exosomes are small membrane-bound vesicles that are secreted when multi-vesicular endosomes fuse with the plasma membrane^3^. They act as cell-to-cell transporters of membrane and cytosolic contents, including proteins, mRNA and microRNA (miRNA); they are also involved in inter-cellular signaling^4^. Exosomes from BMSCs have been shown to provide cardioprotection similar to that seen with the BMSCs as evidenced by decreased anoxia-induced cardiomyocyte apoptosis, increased angiogenesis, increased cardiac function, and decreased infarct size after ischemia/reperfusion injury^4–6^. These cardioprotective effects involve different mechanisms, including suppression of reactive oxygen species and apoptosis that is characteristic of ischemia/reperfusion injury, as well as increasing growth factors that promote angiogenesis, cardiomyocyte proliferation, and tissue repair.

GATA-4 is a cardiomyocyte-specific zinc finger transcription factor that regulates differentiation, growth, and survival^7^. GATA-4-expressing BMSCs improve functional recovery to a greater extent than that observed for BMSCs alone when injected into the myocardium immediately after induction of ischemia^7^. Therefore, we hypothesized that GATA-4-containing exosomes may have a similar effect. In the current study, lentivirus carrying doxycycline (DOX)-inducible *GATA-4* was used to transduce mouse BMSCs, and exosomes released by these BMSCs were collected. The effects of the GATA-4 exosomes on BMSC differentiation into cardiomyocyte precursors and their effects on anoxia-induced apoptosis of cardiomyocytes were investigated using *in vitro* studies. The effect of GATA-4 exosomes on cardiac function was examined in a mouse model of MI. Differential expression of candidate differentiation- and apoptosis-related miRNAs and proteins that may mediate these effects was also identified. Previous studies describing the cardioprotection provided by GATA-4-enriched BMSCs or exosomes have delivered them via intracardial injections before or at the very beginning of ischemia, that growth-promoting actions of GATA-4 is needed, Therefore, our protocol of injecting GATA-4 exosomes at 48 h after initiation of infarction was intended to highlight the actions of GATA-4 on the differentiation and proliferation of cardiomyocyte-like cells.

## Results

### GATA-4 mRNA expression in BMSCs

**Figure 1:**
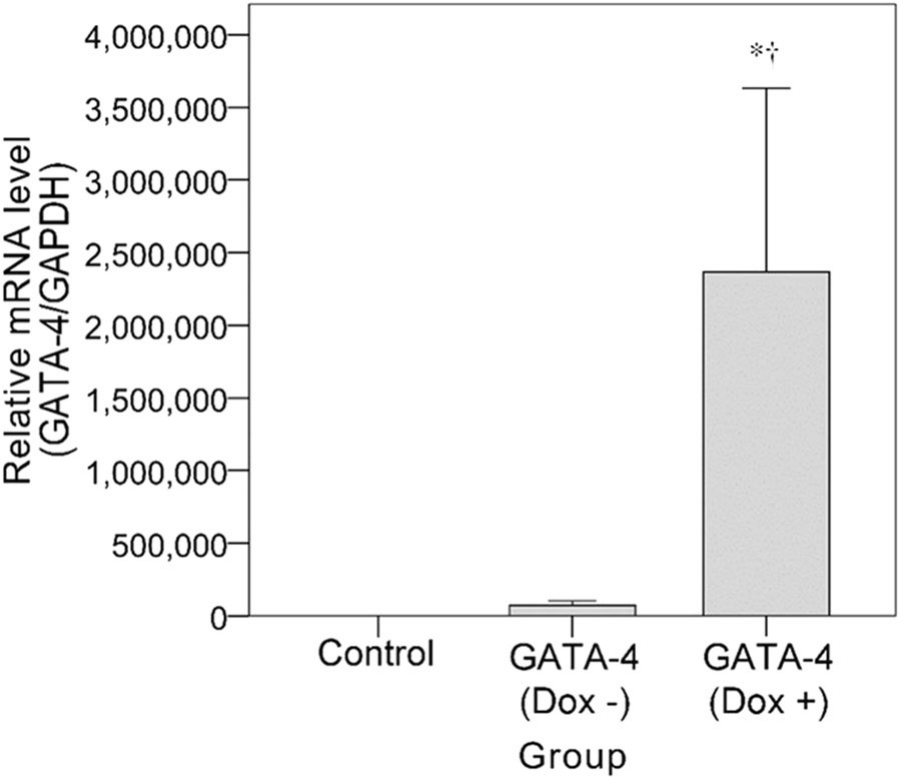
mRNA expression of GATA-4 in BMSC cells. Comparison of GATA-4 mRNA expression in BMSCs transduced with the control virus or GATA-4. Control: no transduction. Transduced: transduction with GATA-4-containing lentivirus. *p < 0.05, significantly different from the control group.

BMSCs were transduced with GV308 lentivirus carrying *GATA-4*. Following induction with DOX, GATA-4 mRNA expression level was further analyzed by real-time RT-PCR. As shown in Fig. 1. GATA-4 mRNA levels are significantly higher following induction by DOX; they were also then that observed in BMSCs transduced with a control lentivirus. Thus, the BMSCs were transduced to express GATA-4 only in the presence of DOX.

### Effect of exosomes secreted by GATA-4-overexpressing BMSCs on the expression of cardiomyocyte markers

**Figure 2:**
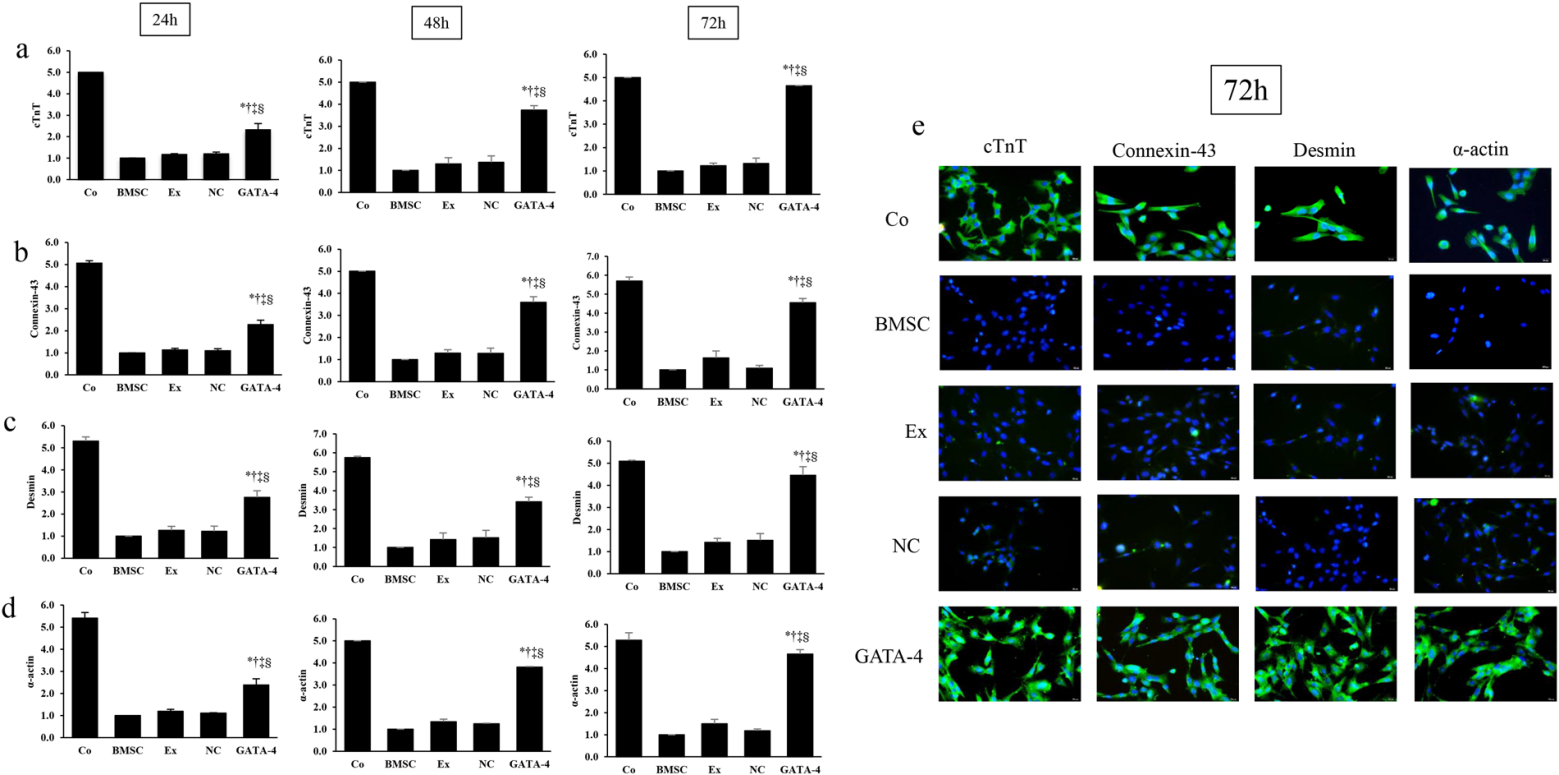
The effect of GATA-4-BMSC exosomes cardiomyocyte marker expression. The mRNA expression of (**a**) cTnT, (**b**) connexin-43, (**c**) desmin, and (**d**) α-actin was analyzed by quantitative PCR in BMSCs following co-culture with GATA-4-BMSC exosomes, NC-BMSC exosomes, BMSC exosomes compared with BMSCs only and cardiomyocyte single cultures. ^*,†,‡,§^*p* < 0.05, significantly different from the ^*^BMSC group, ^†^BMSC exosome group. ^‡^NC-BMSC exosome group, and ^§^cardiomyocyte group. Co, control; BMSC, BMSC only; Ex, exosome BMSC; NC, NC-exosomes; GATA-4, GATA-4-BMSC exosomes.

To examine the effect of exosomes secreted by GATA-4-overexpressing BMSCs on cardiomyocyte transformation by BMSCs, we first examined the expression of the cardiomyocyte-specific antigens, cTnT, connexin-43, desmin and α-actin, following co-culture for 24, 48, and 72 h. As shown in Fig. 2, qPCR analysis showed that cTnT (Fig. 2a), connexin-43 (Fig. 2b), desmin (Fig. 2c), and α-actin (Fig. 2d) levels were significantly higher in co-cultures with exosomes secreted by GATA-4-overexpressing BMSCs (GATA-4 group) compared to those co-cultured with control exosomes or BMSC cultured alone at all time points. Moreover, the expression of each of these markers at 72 h nearly reaches that observed for cardiomyocyte-like cells (Fig. 2e). These data suggest that exosomes secreted by GATA-4-overexpressing BMSCs induce cardiomyocyte-like cell differentiation.

### Effect of exosomes secreted by GATA-4-overexpressing BMSCs on the apoptosis of cardiomyocytes in response to hypoxia

**Figure 3:**
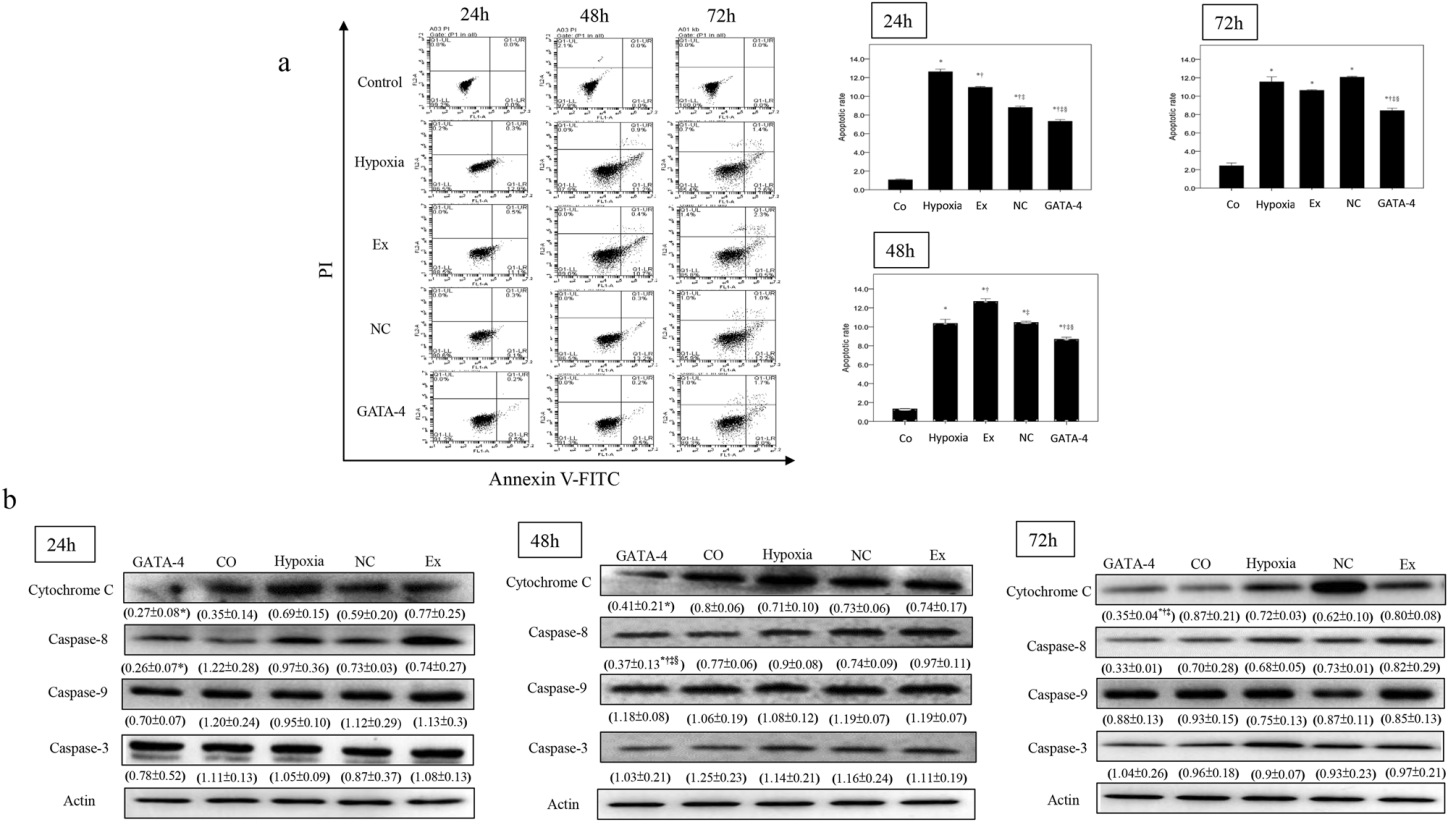
The effect of GATA-4-BMSC exosomes on hypoxia-induced cardiomyocyte apoptosis and caspase expression. (**a**) Cardiomyocyte apoptosis in response to hypoxia and co-culture with exosomes BMSCs, NC-BMSC, or GATA-4 BMSC exosomes were determined by flow cytometry. (**b**) Western blot analysis of cytochrome C, caspase-3, caspase-8, and caspase-9. Relative expression was determined following normalization to actin levels. ^*,†,‡,§^*p* < 0.05, significantly different from the ^*^hypoxia group, ^†^Ex exosome group. ^‡^NC-BMSC exosome group, and ^§^cardiomyocyte group. Co, cardiomyocyte; hypoxia, hypoxia and serum free condition, Ex; exosome BMSC; NC, NC-exosomes; GATA-4, GATA-4-BMSC exosomes.

We next aimed to examine the anti-apoptotic effect of exosomes secreted by GATA-4-overexpressing BMSCs in response to hypoxia, a condition which mimics MI *in vitro*. The rate of hypoxia-induced cardiomyocyte apoptosis was examined by flow cytometry. As shown in Fig. 3a, the rate of cardiomyocyte apoptosis was increased under hypoxia and serum free conditions as compared to the control group cultured under normal conditions. After co-culturing the cardiomyocytes with exosomes secreted by GATA-4-BMSCs, the apoptotic rate was decreased at 24, 48, and 72 h, suggesting that GATA-4-BMSCs suppressed hypoxia-induced cardiomyocyte apoptosis.

Western blot analysis was next undertaken to evaluate the expression of apoptosis-related proteins (Fig. 3b). Under hypoxia and serum free conditions, the expression of caspase 8 was significantly lower in the GATA-4-BMSC group compared to the NC-BMSC group at 24 h and 48 h. The expression of cytochrome C was also significantly lower in GATA-4-BMSC group compared to the NC-BMSC group at 24, 48, and 72 h. No differences in caspase 3 or caspase 9 were noted among the groups. Taken together, these results suggest that exosomes secreted by GATA-4-overexpressing BMSCs can suppress cardiomyocyte apoptosis in response to hypoxia *in vitro*.

### Effect of exosomes secreted by GATA-4-overexpressing BMSCs on cardiac function following MI in mice

**Figure 4:**
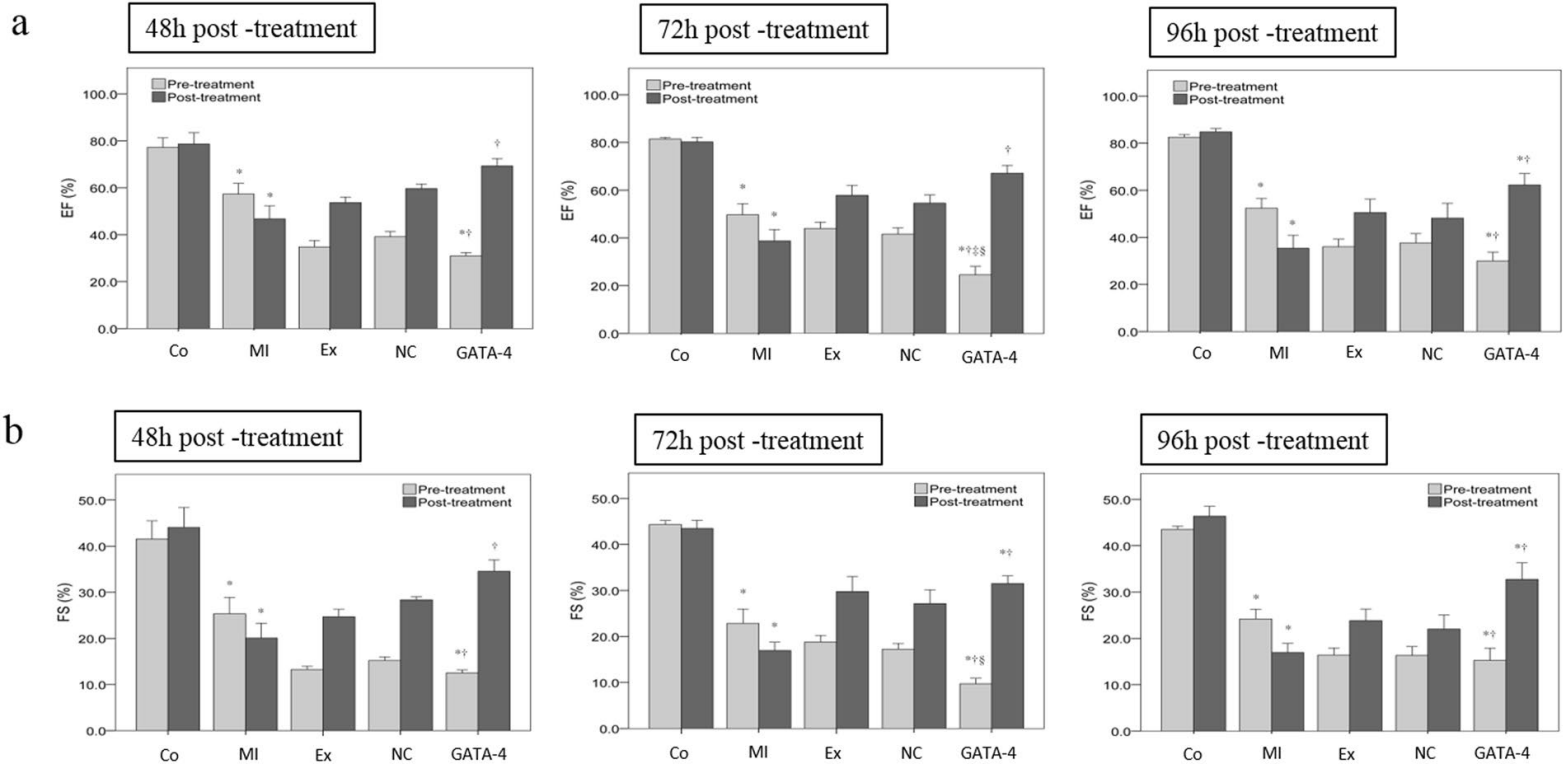
Effect of GATA-4-BMSC exosomes on cardiac function following MI in mice. Differences in (**a**) EF and (**b**) FS following MI. Mice were evaluated for cardiac function 48 h after induction of MI (pre-treatment). The mice were then divided into different groups and injected in the tail vein with GATA-BMSC exosomes, NC-BMSC exosomes, or BMSC exosomes. Cardiac function was then assessed by echocardiography at 48, 72, or 96 h after injection of exosomes (post-treatment). Values for mice without MI are also shown (normal). ^*,†,‡,§^*p* < 0.05, significantly different from the ^*^MI group, ^†^Ex exosome group. ^‡^NC-BMSC exosome group, and ^§^cardiomyocyte group. Co, control; MI, myocardial infarction; Ex, exosome BMSC; NC, NC-exosomes; GATA-4, GATA-4-BMSC exosomes.

Because our previous *in vitro* data suggested that exosomes secreted by GATA-4-overexpressing BMSCs promoted BMSC differentiation into cardiomyocyte-like cells and protected against hypoxia-induced cardiomyocyte apoptosis, we evaluated their effect on cardiac function following induction of MI in mice. As shown in Fig. 4a, the ejection fraction (EF) was significantly decreased in MI treatment group as compared to the normal group (57.32 ± 10.11 versus 77.20 ± 9.12, respectively). Mice were injected in the tail vein with GATA-4-BMSC exosomes at 48 h after induction of MI. We showed that EF was significantly increased as compared to the MI groups at 48 h, 72 h, and 96 h after injection of GATA-4-BMSC exosomes. Similarly, the mean fraction shortening (FS) difference was significantly reduced in the MI group as compared to the normal group (25.34 ± 7.87 versus 41.56 ± 8.73, respectively) at 48 h after exosome treatment. The FS increased in the GATA-4-BMSC exosome group as compared with the normal and MI groups at all time points analyzed (Fig. 4b). Thus, exosomes secreted by GATA-4-overexpressing BMSCs can improve cardiac function *in vivo* following MI.

### Exosomes secreted by GATA-4-overexpressing BMSCs increased exosome intensity in the heart

**Figure 5:**
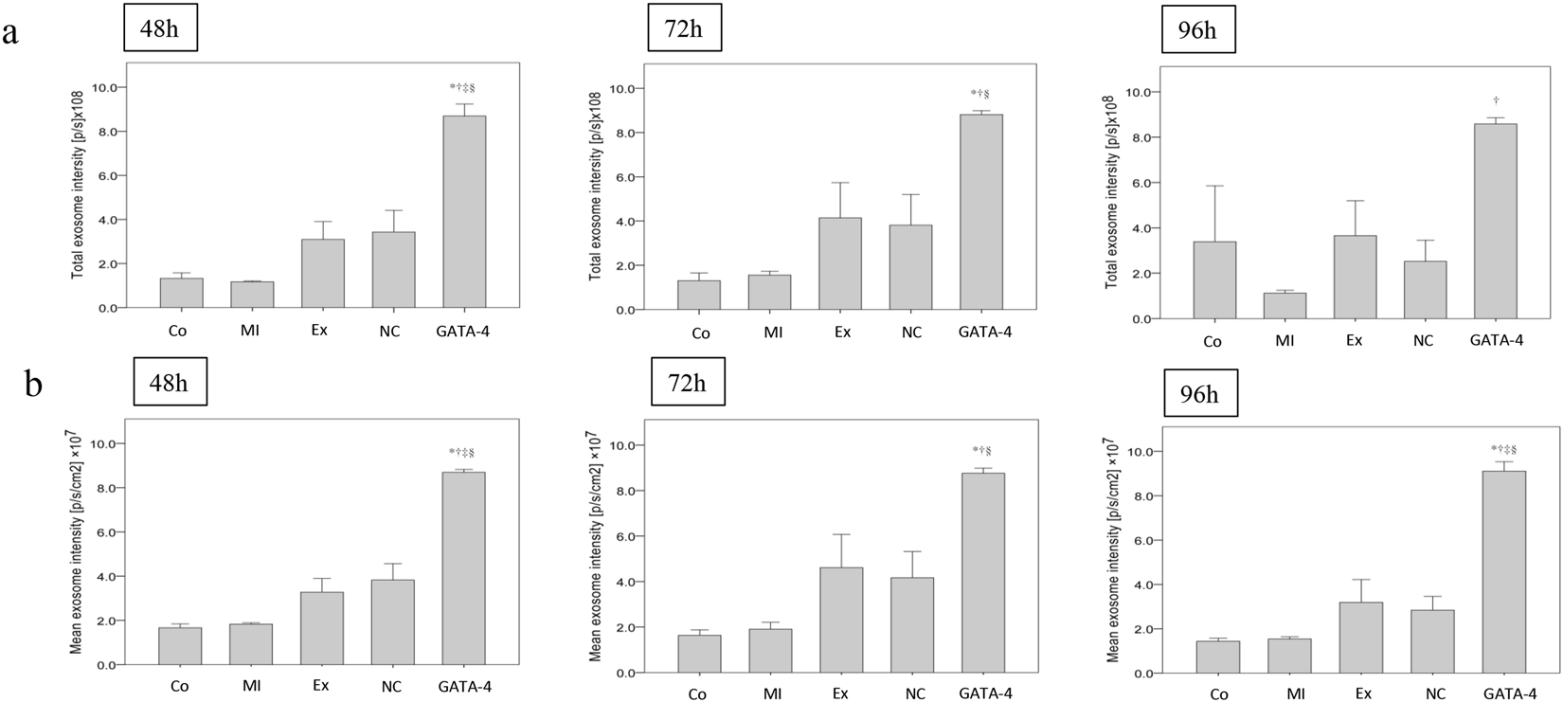
Analysis of exosome homing to cardiac tissue following tail vein injection in an *in vivo* mouse MI model. (**a**) Total exosome intensity and (**b**) mean exosome intensity in the cardiac tissues of mice that were untreated or treated with tail vein injections of GATA-BMSC exosomes, NC-BMSC exosomes, or BMSC exosomes. Values for mice without MI are also shown (normal). ^*,†,‡,§^*p* < 0.05, significantly different from the ^*^normal (N) group, ^†^MI group, ^‡^BMSC-exosome group and ^§^NC-BMSC exosome group. Co, control; MI, myocardial infarction; Ex, exosome BMSC; NC, NC-exosomes; GATA-4, GATA-4-BMSC exosomes.

We next evaluated the levels of exosomes in the cardiac tissues of each group to confirm that some migrated to the cardiac tissue following tail vein injection. As shown in Fig. 5a, the total exosome intensity was significantly higher in the GATA-4-BMSC exosome group as compared to the NC-BMSC exosome group, the BMSC exosome group (nontransduced), the MI group, and the normal group at 48 h. In addition, the mean exosome intensity was significantly higher in the GATA-4-BMSC exosome group as compared to the NC-BMSC exosome, BMSC exosome, MI, and normal groups at all time points analyzed (Fig. 5b). These data confirm that tail vein injections of exosomes secreted by GATA-4-overexpressing BMSCs can migrate to damaged cardiac tissue.

### Effect of exosomes secreted by GATA-4-overexpressing BMSCs on cardiac blood vessel number, apoptotic cell number, and c-kit-positive cell number in MI mice

**Figure 6:**
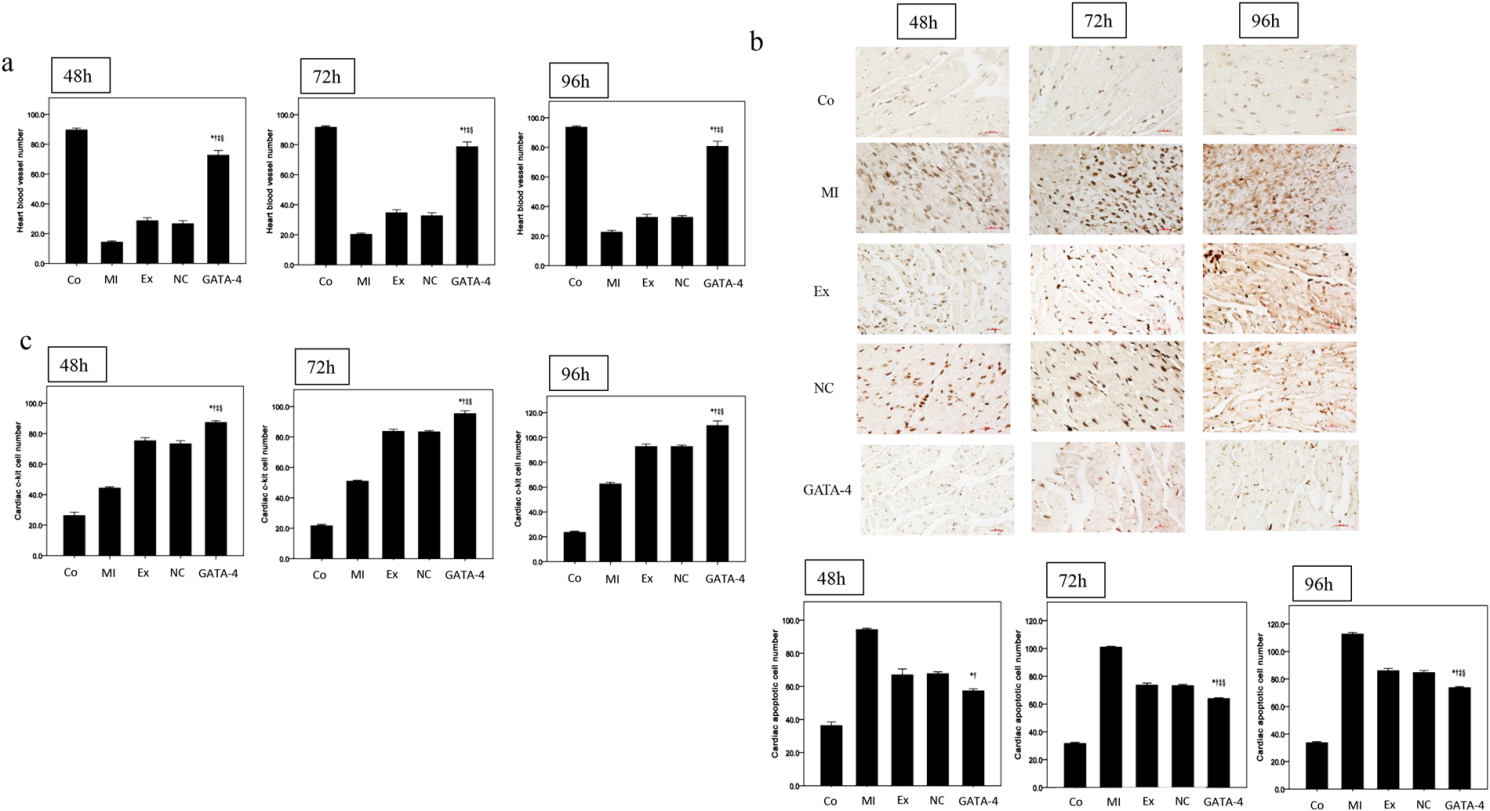
Effect of exosomes secreted by GATA-4-overexpressing BMSCs on cardiac blood vessel number, apoptotic cell and c-kit-positive cell number in MI mice. Following induction of MI, the (**a**) heart blood vessel number, (**b**) apoptotic cell (TUNEL assay), and (**c**) cardiac c-kit cell number were determined in mice that were untreated or treated with tail vein injections of GATA-BMSC exosomes, NC-BMSC exosomes, or BMSC exosomes. Values for mice without MI are also shown (normal). ^*,†,‡,§^*p* < 0.05, significantly different from the ^*^control group, ^†^MI group, ^‡^BMSC-exosome group and ^§^NC-BMSC exosome group. Co, control; MI, myocardial infarction; Ex, exosome BMSC; NC, NC-exosomes; GATA-4, GATA-4-BMSC exosomes.

We next determined the impact of GATA-4 overexpressing BMSCs on cardiac tissue integrity by analyzing the number of blood vessels, apoptotic cells, and c-kit-positive cells (cardiac endothelial cells marker). As shown in Fig. 6a, the mean heart blood vessel number was significantly higher in the GATA-4-BMSC exosome group following MI as compared with the NC-BMSC exosome, BMSC exosome, and MI groups. In addition, the mean cardiac apoptotic cell following MI was significantly lower in the GATA-4-BMSC exosome group as compared with the NC-BMSC exosome, BMSC exosome, and MI groups at 72 and 96 h (Fig. 6b). Finally, the mean number of cardiac c-kit-positive cells was significantly higher in the GATA-4-BMSC exosome group as compared to the NC-BMSC exosome, BMSC exosome, MI, and normal groups at all time points analyzed (Fig. 6c). Taken together, exosomes secreted by GATA-4-overexpressing BMSCs can increase cardiac blood vessel density and the number of c-kit-positive cells as well as decrease the number of apoptotic cardiomyocytes.

### Altered expression of miRNAs associated with cell differentiation and apoptosis protection in exosomes derived from GATA-4-overexpressing BMSCs

To examine the possible miRNAs, which may be involved in the cell differentiation and anti-apoptosis responses by exosomes from GATA-4-overexpressing BMSCs, agilent miRNA chip analysis was undertaken. As shown in Table 1, the top 20 upregulated and downregulated miRNAs and some of their putative functions related to differentiation and apoptosis were identified. Thus, miRNAs associated with differentiation and apoptosis were altered following GATA overexpression in BMSCs.

**Table 1:** Top 20 upregulated and downregulated miRNAs expressed by BMSCs exposed to GATA-4-BMSC exosomes.

Systematic name	FC (abs)	Regulation	Function
mmu-miR-6981-5p	423.2665	up	
mmu-miR-330-3p	183.7133	up	Apoptosis
mmu-miR-6948-3p	154.5511	up	
mmu-miR-7028-5p	137.6545	up	
mmu-miR-7039-5p	125.3424	up	
mmu-miR-7668-3p	124.1184	up	
mmu-miR-3106-5p	121.5087	up	
mmu-miR-122-5p	80.21135	up	Apoptosis
mmu-miR-1929-5p	80.0773	up	
mmu-miR-673-5p	62.80801	up	Differentiation
mmu-miR-378b	61.25606	up	
mmu-miR-98-5p	54.0384	up	Apoptosis
mmu-miR-320-3p	52.96949	up	Apoptosis
mmu-miR-1894-3p	52.54453	up	
mmu-miR-652-5p	45.75114	up	
mmu-miR-7019-5p	44.29345	up	
mmu-miR-1982-5p	40.53661	up	
mmu-miR-28a-5p	39.16947	up	Apoptosis
mmu-miR-6385	36.8192	up	
mmu-miR-130b-3p	36.31932	up	Apoptosis
mmu-miR-7013-3p	111.4338	down	
mmu-miR-6952-3p	99.58505	down	
mmu-miR-6998-3p	91.31043	down	
mmu-miR-7056-3p	89.48467	down	
mmu-miR-2183	88.49762	down	
mmu-miR-6917-3p	88.48234	down	
mmu-miR-669i	86.8465	down	
mmu-miR-764-5p	85.09634	down	Differentiation and apoptosis
mmu-miR-7b-3p	85.02689	down	
mmu-miR-7224-3p	81.42917	down	
mmu-miR-6943-3p	80.84257	down	
mmu-miR-3092-3p	78.4776	down	
mmu-miR-7012-3p	72.97984	down	
mmu-miR-7039-3p	72.892	down	
mmu-miR-675-3p	68.96394	down	
mmu-miR-7087-3p	67.8283	down	
mmu-miR-184-5p	63.7759	down	
mmu-miR-7070-3p	62.87363	down	
mmu-miR-6960-3p	62.51293	down	
mmu-miR-211-5p	61.52079	down	Apoptosis

### Identification of proteins in exosomes secreted by GATA-4-overexpressing BMSCs that are related to cell differentiation and suppression of apoptosis

TMT-labeled quantitative proteomic analysis was next carried out to identify cell differentiation- and anti-apoptosis-related proteins expressed by exosomes secreted by GATA-4-overexpressing BMSCs. Table 2 shows proteins that were differentially expressed in the GATA-4-BMSC exosomes as compared to both the NC-BMSC exosomes and BMSC exosomes, including eight proteins involved in cell differentiation (collagen alpha-1(IV) chain, slit homolog 2 protein, matrilin-2, connective tissue growth factor, phosphatidylethanolamine-binding protein 1, sulfated glycoprotein 1, transferrin receptor protein 1, and tenascin) and six proteins involved in apoptosis (matrix metalloproteinase-9, slit homolog 2 protein, neutrophil gelatinase-associated lipocalin, connective tissue growth factor, macrophage migration inhibitory factor, peroxiredoxin-5, mitochondrial). Moreover, both protein and connective tissue growth factor protein are both involved in apoptosis as well as cell differentiation (Table 2). Therefore, GATA overexpression in BMSCs alters the expression of proteins associated with cell differentiation and apoptosis.

**Table 2:** Candidate cell differentiation- and apoptosis-related proteins differentially expressed by GATA-4-BMSC exosomes.

Protein accession	Protein description	Gene name
P02463	Collagen alpha-1(IV) chain	*Col4a1*
Q9R1B9	Slit homolog 2 protein	*Slit2*
O08746	Matrilin-2	*Matn2*
P29268	Connective tissue growth factor	*Ctgf*
P70296	Phosphatidylethanolamine-binding protein 1	*Pebp1*
Q61207	Sulfated glycoprotein 1	*Psap*
Q62351	Transferrin receptor protein 1	*Tfrc*
Q80YX1	Tenascin	*Tnc*
P41245	Matrix metalloproteinase-9	*Mmp9*
Q9R1B9	Slit homolog 2 protein	*Slit2*
P11672	Neutrophil gelatinase-associated lipocalin	*Lcn2*
P29268	Connective tissue growth factor	*Ctgf*
P34884	Macrophage migration inhibitory factor	*Mif*
P99029	Peroxiredoxin-5, mitochondrial	*Prdx5*

## Discussion

Exposure of BMSCs to exosomes collected from GATA-4 transduced BMSCs in an anoxic environment induced their differentiation cardiomyocyte precursors. GATA-4-BMSC exosomes also decreased apoptosis in cardiomyocytes subjected to anoxia. In addition, when injected into the tail vein of mice 48 h after MI, GATA-4-BMSC exosomes significantly improved cardiac function over the next 96 h with increased cardiac vessel density and number of c-kit-positive cells while decreasing cardiomyocyte apoptosis *in vivo*. In contrast, control exosomes did not induce BMSC differentiation, had a marginal effect in decreasing cardiomyocyte apoptosis, and had no effect on post-infarction ventricular function.

*GATA-4* is a cardiac gene, and GATA-4 mRNA was only detected in BMSCs transduced with GATA-4 lentivirus, but not in BMSCs transduced with lentivirus alone, confirming that baseline GATA-4 levels are extremely low in BMSCs. GATA-4 plays an important role in the early development of the heart, and increasing its expression promotes the differentiation of precursor cells into cardiomyocytes^8,9^. Although GATA-4-BMSC exosomes increased cardiomyocyte-like differentiation of BMSCs, it was likely through a mechanism different from BMSC differentiation into other cell types, which does not involve GATA-4^1,10^. In the present study, differential expression of candidate differentiation-related miRNAs and proteins that may mediate these effects was also identified. However, the exact mechanism by which GATA-4-BMSC exosomes induce myocyte differentiation by BMSCs remains unknown and will be the focus of future studies. The influence of the identified miRNAs and proteins as potential mediators of GATA-4-induced differentiation will also be assessed.

In our study, GATA-4-BMSC exosomes decreased apoptosis in cardiomyocytes subjected to anoxia to a greater extent than control exosomes. Decreased *in vivo* apoptosis following injection of GATA-4-BMSC exosomes was also shown. These results are similar to those of a previous study by Yu *et al*.^11^, which suggested that GATA-4 expression in BMSCs caused them to produce anti-apoptotic miRNAs that were transferred to hypoxic cardiomyocytes via exosomes, resulting in the activation of the Akt and ERK pathways. Specifically, exosomes from GATA-4-transduced BMSCs were rapidly internalized by cardiomyocytes that had been exposed to hypoxia and reduced apoptosis in these cells^11^. Thus, it is possible that the anti-apoptotic effects observed in the present study worked through a similar mechanism; however, further studies are necessary to confirm this. Further studies will also examine the role of the differentially proteins as potential mediators of GATA-4-induced protection from apoptosis.

Another study suggested that anti-apoptotic miRNA may be effective in preventing ischemia/reperfusion damage only if injected at the beginning of ischemia, the period in which inflammatory cytokines and an increase in reactive oxygen species cause mitochondrial damage and subsequent apoptosis^3^. MiR-146a is a GATA-4-responsive anti-apoptotic miRNA that negatively regulates the TLR-mediated NF-κB pathway and decreases inflammatory cytokines^12,13^. MiR-146a is increased in cardiomyocyte-derived exosomes and in MI tissue that has been exposed to these exosomes at the beginning of ischemia. These cardiomyocyte-derived, miR-146a-containing exosomes increased LVEF after MI when injected either at the initiation of the infarction (i.e., before ischemia) or 3 weeks later. Injection of the anti-apoptotic miRNA itself, however, only increased LVEF when given at the initiation of the infarction and had no effect on this parameter when given later^3^. Although miR-146a was not identified as differentially expressed in the present study, several other miRNAs with roles in apoptosis were detected; therefore, further studies will be undertaken to analyze their role in mediating the effects of GATA-4-BMSC exosomes.

Previous studies have shown that exosomes from MSCs that had not been transduced with GATA-4 could increase LVSF and FS several weeks after induction of MI^4,11,14^, which is in contrast with the present study. MSCs are known to secrete cytokines, chemokines, and growth factors^5^, and contain an anti-inflammatory miRNA, including miR-223, that has been shown to be partly responsible for the increased cardiac function^14^. Exosomes derived from GATA-4 transduced MSCs have been called a “reservoir” for anti-apoptotic miRNAs^11^, but they also contain growth factors, and miR-233, which regulates cardiomyocyte proliferation^14^. Thus, further studies are necessary to characterize the GATA-4-exosomes for both growth factor and miRNA levels. The difference between our study and those studies in which both GATA-4-exosomes and control exosomes improved post-infarction ventricular function may be due to the timing of the exosome injections (i.e., at the beginning of infarction versus at 48 h after infarction). Our previous data showed a significant difference in EF and FS between the infarct group and the control group at 48 h after induction of MI. The first 48 hours after induction of infarct in mice is a very critical period when there may be death due to excessive ligation or infection. A successful MI model is therefore defined as survival beyond the initial 48 h post-induction period. In our present study, mice were treated with exosomes after confirming that cardiac function was affected, and the effect of exosome treatment on cardiac function was assessed at 48 h, 72 h and 96 h after exosome treatment. Our results suggest that exosomes from MSCs and BMSCs transduced with GATA-4 both have anti-apoptotic activity, but GATA-4-exosomes may also induce cardiomyocyte-specific proliferative activity.

The present study has some limitations that warrant further discussion. For example, cardiomyocyte apoptosis was determined by TUNEL assay. Because the involvement of other transcription factors, growth factors, mRNA, and non-coding regulatory miRNA cannot be ruled out, further studies will examine their putative roles in cardiac function improvement following MI.

In the present study, exosomes isolated from GATA-4-expressing BMSCs improved cardiac function as well as tissue integrity when injected 48 h after initiation of infarction in mice. These effects may be mediated by the anti-apoptotic and proliferative effects of these exosomes on cardiomyocytes.

## Materials and Methods

### Establishment and identification of mouse BMSC cultures

Mouse BMSCs were obtained from the femur and tibia of five healthy male C57BL/6 mice sacrificed by cervical dislocation as previously reported with modification^15^. Animals were housed and maintained according to the regulations for the Non-clinical Research Management Practice. This study was approved by the Institutional Animal Care and Use Committee of the First People’s Hospital of Yunnan Province. The isolated cells were re-suspended in bone marrow mesenchymal stem cell growth medium (Cyagen Biosciences, Sunnyvale, CA, USA) containing 10% fetal bovine serum, seeded into a 25-cm^2^ flask at a density of 2 × 10^6^/cm^2^, and incubated at 37 °C and 5% CO_2_. These cells are at Passage 0 (P0). Flow cytometry was used to detect the levels of cell surface markers on BMSCs in P9 using the following antibodies: rabbit anti-mouse FITC-conjugated CD11b, anti-mouse/human PE/Cy5-conjugated CD44, anti-mouse/rat PE-conjugated CD29, and anti-mouse/PE-conjugated Sca-1 antibodies (BioLegend, San Diego, CA, USA). Rabbit anti-mouse IgG-FITC served as an isotype control. P9 cells were selected for all subsequent experiments because the expression of surface makers and cell growth are optimal at this time.

### Transduction of BMSCs with lentivirus carrying GATA-4

GV308 lentivirus carrying *GATA-4* was used to transduce BMSCs using polybrene as the linker molecule. RT-PCR was employed to confirm that BMSCs express very low levels of endogeous GATA-4. Cells with successful transduction showed green fluorescence. The reaction mixture contained dNTP mix (2.5 mM each), forward and reverse primers (10 µM each; Shanghai Genechem Co., Ltd., Shanghai, China), template (10 ng/µL), and PrimeSTAR HS DNA polymerase (0.5 µL).

When the transduction efficiency reached 80%, the multiplicity of infection (MOI) was determined. The required MOI was 20–50. Transduction with the lentivirus vector was also performed as a control using the same protocol.

### Extraction of exosomes from lentivirus-transduced BMSCs

Removal of serum exosomes was achieved by ultra-centrifugation at 150,000 × g and 4 °C overnight. The processed serum used for subsequent culture experiments. GATA-4 transuced BMSC cells were incubated with processed serum and culture medium for 48 hours with 5 μg/mL of doxycyclin addition at the same time. The secreted exosomes were furthered extracted by EXOTC50A-1 ExoQuick TC (SBI, SBI, Mountain View, CA) according to manufacturer’s protocol.

### Co-culture of exosomes and BMSCs

P9 BMSCs were co-cultured in a serum free, hypoxic environment (5% oxygen for 24 h) with exosomes from control lentivirus-transduced and GATA-4-lentivirus-transduced BMSCs. BMSCs not exposed to exosomes were cultured as another control group. For exosome co-cultures, BMSCs were added to a 25-cm^2^ flask, followed by addition of exosomes at 20 µg/mL. After 14 days, the expression of myocyte-specific markers (e.g., α-actin, cTnI, Cx43, and desmin) and GAPDH was measured by immunofluorescence analysis and quantitative PCR (qPCR)^16^. Briefly, total RNA was extracted using Trizol (Invitrogen, Carlsbad, CA, USA). cDNA was synthesized using AMV First Strand cDNA Synthesis Kit (New England Biolabs, Ipswich, MA, USA), and qPCR was performed using an ABI StepOne plus as manufacture’s protocol. The reaction mixture contained SybrGreen qPCR Master Mix, primers (10 µM each; Tables 1 and 2), and double-distilled water in a total volume of 20 µL.

### Co-culture of exosomes and cardiomyocytes

One mL of exosomes (in culture medium; 20 µg/mL) from control lentivirus-transduced or GATA-4 lentivirus-transduced BMSCs were co-cultured with 1 × 10^5^ C57BL/6 mouse ventricular cardiomyoctes (ScienCell, San Diego, CA, USA), which express very low levels of endogeous GATA-4 (data not shown), in a hypoxic, serum-free environment.

### Quantitative PCR analysis

The quantitative PCR was performed as previously described^17^. At 24, 48, and 72 h after hypoxia, the expression of cTnT, connexin-43, desmin, and α-actin in BMSCs was analyzed by quantitative PCR. The primer sequences were listed in Table 3.

**Table 3:** Primer sequences in this study.

Name of Genes		Sequence
cTnT	F′	AATGAAGACCAACTGAGA
	R′	TATTTCTGCTGCTTGAAC
Connexin-43	F′	TCAAGAAGTTCAAGTATGG
	R′	ATGCTGATGATGTAGGTT
Desmin	F′	CGTGACAACCTGATAGAC
	R′	TTCTCTGCTTCTTCTCTTAG
α-actin	F′	GAGTAATGGTTGGAATGG
	R′	GTTCTATCGGATACTTCAG
GAPDH	F′	TGGTGAAGGTCGGTGTGAAC
	R′	GCTCCTGGAAGATGGTGATGG
GATA-4	F′	CGAGGGTGAGCCTGTATGT
	R′	TGCTGTGCCCATAGTGAGAT

### Western blot analysis

Following incubation with cell lysis buffer (Shanghai Beyotime Biotechnology Institute, Shanghai, China), 70 µg of protein was loaded for separation by SDS-PAGE. After the proteins were transferred to a PVDF membrane, the membranes were blocked with 5% BSA and incubated in the following antibodies: anti-caspase-3 (1:1000; Cell Signaling Technology, Danvers, MA, USA), anti-caspase-8 (1:1000; Cell Signaling Technology), anti-caspase-9 (1:1000; Cell Signaling Technology), and cytochrome C (1:1000; Cell Signaling Technology) antibodies. The membranes were subsequently incubated with HRP-conjugated secondary antibodies (1:5000; sc-2005; Santa Cruz Biotechnology, Santa Cruz, CA, USA), and ECL (Millipore Temecula, CA, USA) was used to visualize the bands. The relative protein expression was determined following normalization with actin.

### Flow cytometry

Flow cytometry was performed as previously described^18^. Cells were harvested at various time points after they were harvested with trypsin-EDTA, washed with PBS, and fixed in 70% ethanol at 4 °C for 1 h. Ethanol was removed, and the cell pellet was washed with PBS twice before staining. An Annexin-V apoptosis kit (Invitrogen) was used for the detection of cell apoptosis using a BD FACS Celesta flow cytometer (BD Biosciences, Franklin Lakes, NJ, USA). Briefly, propidium iodide (PI) fluorescence was analyzed by excitation with a 488 nm argon ion laser and detection using a 620 nm band-pass filter. Doublets and higher aggregates were excluded by gating, and 10,000 events (excluding doublets) were recorded per sample.

### Establishment of a MI model

MI was induced in mice according to previous reports with modifications^17^. Briefly, mice were intraperitoneally anesthetized with 10% chloral hydrate at 1.5 mL/kg, fixed on a table in a supine position, and the four limbs were connected to an ECG monitor. Mice were then intubated and ventilated with a small animal respirator. Thoracotomy was performed, and the heart was exposed through pericardiotomy. An 8.0 suture was placed in the myocardium under the left anterior descending artery at 1–2 cm below the root of the left atrial appendage. Reduced movement of the left ventricular wall and apex indicated successful ligation. The ECG after ligation showed clear elevation of the S-T segment (0.2 mm elevation in S-T segment in lead II). After surgery, mice were intraperitoneally injected with penicillin (50,000 U) to prevent infection. The establishment of the MI model was confirmed by pathology, ECG, and Doppler echocardiography. *In vivo* apoptosis was determined using the TUNEL assay (Roche, Indianapolis, IN, USA) following the manufacturer’s instructions.

### Echocardiography assessment of ventricular function and the determination of heart fluorescence intensity

The echocardiography assessment was performed as previously described^17^. At 48 h following induction of MI as well as at 48 h, 72 h and 96 h after exosome injection, Doppler echocardiography with a Philips IE33 ultrasound machine was performed to evaluate cardiac function. M-mode echocardiography was performed simultaneously. Left ventricular fractional shortening (FS) and ejection fraction (EF) were measured in three cardiac cycles. After echocardiography, the mice underwent small animal *in vivo* imaging (Caliper IVIS Lumina LT), and the heart fluorescence intensity was further determined.

### Exosome administration after MI

MI was induced in C57BL/6 mice, and the mice were evaluated after 48 h to assess their cardiac function (pre-treatment time point). The mice were then divided into three groups and injected in the tail vein with different exosomes at 48 h after induction of MI (n = 5/group): GATA-4-BMSC exosomes (exosomes from GATA-4-expressing BMSCs; 1 mL; 20 μg/mL), NC-BMSC exosomes (exosomes from BMSCs without GATA-4 transduction; 1 mL; 20 μg/mL), and no exosomes (1 mL of saline). Cardiac echocardiography was performed in three mice from each of the three groups to evaluate cardiac function before exosome injection (pre-treatment) and at 48 h,72 h and 96 h after exosome injection (post-treatment).

### Using Agilent miRNA chip analysis

In the presence of DOX, BMSCs overexpressing GATA-4 and control BMSCS were maintained in serum-containing medium without exosomes for at least 48 h. The ExoQuick-TC method was employed to extract the exosome total RNA and purified miRNA with a mirVana™ miRNA Isolation Kit (AM1561) following the manufacturer’s instructions. After 200 ng of total RNA was purified, it was labeled with an Agilent miRNA Complete Labeling and Hyb Kit (Richardson, TX, USA). Agilent Feature Extraction (v10.7) was used to analyze the images after hybridization, followed by data extraction. Proteins were considered to be upregulated at a ratio of >1.2 and downregulated at a ratio of <0.83, Agilent GeneSpring software was used for the data normalization. GeneSpring was used for the analysis of intergroup difference.

### Quantitative proteomic analysis

BMSCs were incubated with lysis buffer (8 M urea, 1% TritonX-100, 65 mM DTT, 1% Protease inhibitor, 3 μM TSA, 50 mM NAM, and 2 mM EDTA), followed by sonication on ice. After centrifugation at 4 °C for 10 min at 12,000 × g, cell debris was removed, and the supernatant was collected and incubated with 15% TCA at 4 °C for 2 h. Following centrifugation at 4 °C for 3 min at 12,000 × g, the supernatant was removed, and the sediment was washed in pre-cold acetone. After the sediment was dissolved in a urea solution (8 M urea, 100 mM TEAB, pH8.0), the protein solution was mixed with DTT at a final concentration of 10 mM followed by incubation at 37 °C for 1 h. After 20 mM IAA was added for 45 min in the dark at room temperature, the urea was diluted to lower than 2 M and the TEAB concentration was as high as 100 mM. Trypsin was added at a ratio of 1:50 (w:w; trypsin:protein) for an overnight incubation after which it was added at a ratio of 1:100 (w:w; trypsin:protein) for 4 h. After treatment with Strata X C18 (Phenomenex) followed by freeze-drying in a vacuum, 0.5 M TEAB was added to dissolve the peptides, which were labeled according to the manufacturer’s instructions (6-TMT kit, Thermo Scientific, IL, USA). Peptides were then processed by reverse phase HPLC fractionation with high pH (Agilent 300Extend C18 column; 5 μm particles, 4.6 mm ID, 250 mm length). The peptides were further validated by LC-MS/MS analysis.

### Statistical analysis

Data were presented as means and standard deviations (SDs), and one-way ANOVA with Bonferroni post-hoc tests were performed to compare the differences between different groups. For the miRNA chip analysis, comparisons of differentially expressed genes were performed with t-tests between two groups and ANOVA among groups. Statistical analyses were performed by IBM SPSS statistical software version 22 for Windows (IBM Corp., Armonk, NY, USA) A two-tailed *p*-value < 0.05 indicated statistical significance.
