# Author Correction: GATA-4-expressing mouse bone marrow mesenchymal stem cells improve cardiac function after myocardial infarction via secreted exosomes

**DOI:** 10.1038/s41598-023-29697-4

**Published:** 2023-02-16

**Authors:** Ji-Gang He, Hong-Rong Li, Jin-Xiu Han, Bei-Bei Li, Dan Yan, Hong-Yuan Li, Ping Wang, Ying Luo

**Affiliations:** 1 Department of Cardiac and Vascular Surgery, First People’s Hospital of Yunnan Province, No. 157 Jinbi Road, Kunming, 650032 Yunnan Province China; 2 Kunming University of Science and Technology, NO.68, Wenchang Road, 121 Street, Kunming, 650032 Yunnan Province China

Correction to: *Scientific Reports* 10.1038/s41598-018-27435-9, published online 13 June 2018

This article contains an error in Figure 2, where the BMSC-cTnT image is a duplication of the NC-Desmin image, and the BMSC-Desmin image is a duplication of the Ex-Desmin image.

The corrected Figure 2 and its accompanying legend appear below.

**Figure 2:**
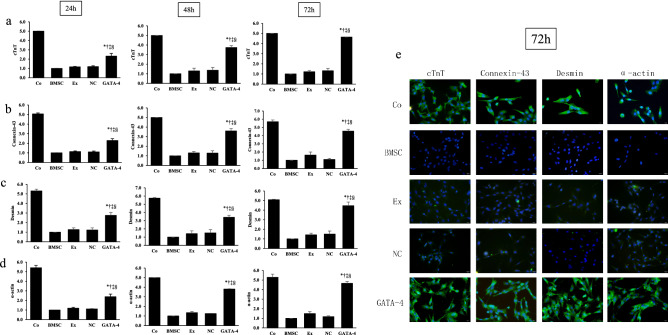
The effect of GATA-4-BMSC exosomes cardiomyocyte marker expression. The mRNA expression of (**a**) cTnT, (**b**) connexin-43, (**c**) desmin, and (**d**) α-actin was analyzed by quantitative PCR in BMSCs following co-culture with GATA-4-BMSC exosomes, NC-BMSC exosomes, BMSC exosomes compared with BMSCs only and cardiomyocyte single cultures. ^*,†,‡,§^*p* < 0.05, significantly different from the *BMSC group, ^†^BMSC exosome group. ^‡^NC-BMSC exosome group, and ^§^cardiomyocyte group. Co, control; BMSC, BMSC only; Ex, exosome BMSC; NC, NC-exosomes; GATA-4, GATA-4-BMSC exosomes.

